# The *in Vitro* Estrogenic Activities of Polyfluorinated Iodine Alkanes

**DOI:** 10.1289/ehp.1103773

**Published:** 2011-10-11

**Authors:** Chang Wang, Thanh Wang, Wei Liu, Ting Ruan, Qunfang Zhou, Jiyan Liu, Aiqian Zhang, Bin Zhao, Guibin Jiang

**Affiliations:** State Key Laboratory of Environmental Chemistry and Ecotoxicology, Research Center for Eco-Environmental Sciences, Chinese Academy of Sciences, Beijing, People’s Republic of China

**Keywords:** endocrine disruptor, estrogenic effects, *in vitro* assay, perfluorinated chemicals, polyfluorinated iodine alkanes

## Abstract

Background: Polyfluorinated iodine alkanes (PFIs) are important intermediates in the synthesis of organic fluoride products. Recently, PFIs have been detected in fluoropolymers as residual raw materials, as well as in the ambient environment.

Objectives: High production volumes and potential environmental releases of PFIs might become a concern, but the exposure risk and toxicity of these chemicals are still unclear. In this study, we investigated the potential estrogenic effects of PFIs.

Methods: We studied the estrogenic effects of fluorinated iodine alkanes (FIAs), fluorinated telomer iodides (FTIs), and fluorinated diiodine alkanes (FDIAs) using the E-screen and MVLN assays and the evaluation of estrogen-responsive genes in MCF-7 cells.

Results: FIAs have an iodine atom at one end of the perfluorinated carbon chain. 1-Iodoperfluorohexane (PFHxI) and 1-iodoperfluorooctane (PFOI) promoted the proliferation of MCF-7 cells, induced luciferase activity in MVLN cells, and up-regulated the expression of *TFF1* and *EGR3*. In these assays, other FIAs gave negative responses. FDIAs have an iodine atom at each end of the perfluorinated carbon chain, and all the FDIAs showed estrogenic effects. The estrogenic potencies of FIAs and FDIAs correlate well with the carbon chain length of the chemicals. The optimum chain length for estrogenic effects is six carbons, and then eight and four carbons. All FTIs have a single iodine atom at the end of a partially fluorinated carbon chain. None of the FTIs showed estrogenic effects in the tests.

Conclusions: The estrogenic effects of PFIs are dependent on the structural features of iodine substitution and chain length. This research will be helpful in further understanding the estrogenic effects of perfluorinated compounds.

Perfluorinated chemicals (PFCs) have a broad range of applications in the manufacture of various industrial and commercial products, such as fluoropolymers, surfactants, emulsifiers, and nonstick coatings. PFCs have been of considerable scientific and public concern because some of them are environmentally persistent, bioaccumulative, and widely detected in humans, wildlife, and the environment (Giesy and Kannan 2001; Olsen et al. 2007).

Because of environmental concerns, the 3M company voluntarily phased out electrochemical fluorination-based fluorochemicals in 2001 (Dupont 2005). Consequently, the current production of fluorinated polymers and surfactant is mostly based on telomerization processes (Lehmler 2005). Polyfluorinated iodine alkanes (PFIs) are organic iodides composed of a fluorinated carbon backbone terminated by iodine substitution (Table 1) and are important intermediates in the synthesis of various fluorinated chemicals (Brace 1999; Prevedouros et al. 2006). In the telomerization process, PFIs are used to synthesize fluorotelomer alcohols (FTOHs) and other related PFCs. In turn, FTOHs are intermediates in the production of surfactants and fluoropolymers, and these volatile compounds have been detected in the atmosphere around the world (Ellis et al. 2004). The annual production of FTOHs increased to 11–13 × 10^3^ metric tons in 2002 (Ellis et al. 2003). The annual world production of PFIs has been estimated to exceed 4,000 metric tons (Organisation for Economic Co-operation and Development 2004), and the increasing demand of fluorotelomer products might increase the risk of emission of volatile PFIs to the environment (Ruan et al. 2010a, 2010b). Fluorinated iodine alkanes (FIAs) and fluorinated telomer iodides (FTIs) have been detected in air and soil samples around a fluorochemical manufacturing plant in Shandong province in northern China (Ruan et al. 2010a). Residual FIAs and FTIs could also be incorporated into FTOH containing raw materials and fluorotelomer-based products during manufacturing. 1-Iodoperfluorooctane (PFOI) and 6:2 FTI have been detected in fluorotelomer raw materials and selected fluorotelomer-based products, such as urethane polymer and phosphate surfactant (Larsen et al. 2006). Furthermore, unreacted residual FTOH has also been found in commercial and industrial products and could be released to the ambient environment as well (Dinglasan-Panlilio and Mabury 2006; Larsen et al. 2006). Likewise, residual PFIs in fluorinated polymers and surfactants can be released into the environment and degrade to other persistent PFCs. Abiotic or biotic transformation of FTIs could contribute to the environmental burden of FTOHs and perfluorocarboxylic acids (PFCAs) (Young et al. 2008). There is therefore a potential risk for release of PFIs to the environment due to direct emission during manufacturing and indirect emission from some fluorinated products.

**Table 1:** The structures of tested chemicals.

Structure	Chemical
Fluorinated iodine alkanes (FIAs)	
	PFBI, 1-iodoperfluorobutane (a = 1)
	PFHxI, 1-iodoperfluorohexane (a = 3)
	PFOI, 1-iodoperfluorooctane (a = 5)
	PFDI, 1-iodoperfluorodecane (a = 7)
	PFDoI, 1-iodoperfluorododecane (a = 9)
Fluorinated telomer iodides (FTIs)	
	4:2 FTI, 1*H*,1*H*,2*H*,2*H*-perfluorohexyl iodide (b = 2)
	6:2 FTI, 1*H*,1*H*,2*H*,2*H*-perfluorooctyl iodide (b = 4)
	8:2 FTI, 1*H*,1*H*,2*H*,2*H*-perfluorodecyl iodide (b = 6)
	10:2 FTI, 1*H*,1*H*,2*H*,2*H*-perfluorododecyl iodide (b = 8)
Fluorinated diiodine alkanes (FDIAs)	
	PFBDI, Octafluoro-1,4-diiodobutane (c = 4)
	PFHxDI, Dodecafluoro-1,6-diiodohexane (c = 6)
	PFODI, Hexadecafluoro-1,8-diiodooctane (c = 8)
	PFOC, 1H-perfluorooctane (R = H)
	PFOB, 1-bromoperfluorooctane (R = Br)
	PFOA, perfluorooctanoic acid
	1-Iodohexane
a, b, and c indicate the number of CF_2_ units.	

Increasing evidence has shown that some PFCs may have endocrine-disrupting potency. Some PFCs can disturb the thyroid system and neuroendocrine function, activate both peroxisome proliferator-activated receptors and estrogen receptors (ERs), and induce developmental toxicity in rodents (Lau et al. 2004). The estrogenic effects of some PFCs have been studied in many aspects. For example, Maras et al. (2006) demonstrated the estrogen-like properties of FTOHs in MCF-7 cells. Using yeast two-hybrid assays, Ishibashi et al. (2007, 2008) demonstrated that FTOHs can activate the male medaka (*Oryzias latipes*) and human ER. Liu et al. (2007) reported that vitellogenin expression was induced by perfluorooctane sulfonate (PFOS), perfluorooctanoic acid (PFOA), and FTOHs in primary cultured tilapia hepatocytes, and they suggested that estrogenic effects may be mediated through the ER pathway. FTOHs also induced vitellogenin in male medaka fish through the activation of ER, whereas PFOS and PFOA did not (Ishibashi et al. 2008).

Little information is currently available regarding the estrogenic effects of PFIs. In the present study, we used three *in vitro* bioassays—E-screen assay, MVLN assay, and evaluation of an estrogen-responsive gene—to comprehensively evaluate the estrogenic potencies of PFIs. The structural features responsible for estrogenic effects were identified by the alternations in potency derived from specific structural changes.

## Materials and Methods

*Chemicals.* The chemical structures of tested compounds are shown in Table 1. We purchased 1-iodoperfluorobutane (PFBI; 98% pure), 1-iodoperfluorohexane (PFHxI; 99% pure), 1-iodoperfluorodecane (PFDI; 97% pure), 1-iodoperfluorododecane (PFDoI; 97% pure), 6:2 FTI (96% pure), 8:2 FTI (96% pure), hexadecafluoro-1,8-diiodooctane (PFODI; 98% pure), 1H-perfluorooctane (PFOC; 99% pure), 1-bromoperfluorooctane (PFOB; 99% pure), 1-iodohexane (98% pure), and 4-hydroxytamoxifen (OHT; 98% pure) from Sigma Chemical Company (St. Louis, MO, USA); PFOI (98% pure), 4:2 FTI (95% pure), 10:2 FTI (95% pure), and PFOA (98% pure) from Fluka (Buchs, Switzerland); and octafluoro-1,4-diiodobutane (PFBDI; 97% pure), dodecafluoro-1,6-diiodohexane (PFHxDI; 97% pure), and 17β-estradiol (E_2_; 99% pure) from Alfa Aesar (Ward Hill, MA, USA). We dissolved all the PFIs, PFOB, E_2_, and 1-iodohexane in ethanol. PFOA and PFOC were dissolved in dimethyl sulfoxide as 100 mM and 10 mM stock solutions. All stock solutions were stored at –20°C.

*Cell culture.* Human MCF-7BUS breast adenocarcinoma cells and MVLN cells were cultured in 100-mm culture dishes in a humidified atmosphere of 5% CO_2_ at 37°C. Cells were maintained in Dulbecco’s modified Eagle’s medium (DMEM)/F-12 (Hyclone, Logan, UT, USA) containing 10% fetal bovine serum, 100 U/mL streptomycin-penicillin, 2 mM l-glutamine, and 1% insulin-transferrin-selenium supplement (all from Gibco, Grand Island, NY, USA).

*E-screen assay.* MCF-7BUS cells were kindly provided by A.M. Soto and C. Sonnenschein (Tufts University School of Medicine, Boston, MA, USA). In response to ERα agonists, the mitotic effect leads to the proliferation of MCF-7BUS cells. We performed the E-screen assay following a method modified from the protocol by Soto et al. (1995). Cells were trypsinized and plated into the interior 60 wells of 96-well plates at the density of 3,000 cells/well. Before each experiment, cells were starved in steroid-free (SF) medium for 48 hr to minimize the basal hormonal activity during assays. SF medium consisted of phenol red–free DMEM/F-12 (Hyclone) supplemented with 5% dextran-charcoal-treated fetal bovine serum (Hyclone), 100 U/mL streptomycin-penicillin, and 2 mM l-glutamine. Cells were treated with serial dilutions of test chemicals (from 1 nM to 100 μM) in SF medium; a concentration range of 0.01–200 pM E_2_ was used as the positive control. We used a WST-1 proliferation kit (Roche Diagnostics, Mannheim, Germany) to assess proliferation after 6 days of exposure according to the kit instructions. The WST-1 assay is based on the enzymatic cleavage of the tetrazolium salt WST-1 to formazan by cellular mitochondrial dehydrogenases present in viable cells. The absorbance of the WST-1 solution was detected by a microplate reader (Varioskan Flash, Thermo Fisher Scientific, Waltham, MA, USA) at 450 nm, with the reference wave length at 690 nm. The cell proliferation effect was calculated from the solvent control (0.1% ethanol)-corrected absorbance and expressed as the percentage of maximal absorbance of the positive control. Three replicates were used in each experiment.

*MVLN assay.* The MVLN cell line was kindly provided by J.P. Giesy (Michigan State University, East Lansing, MI, USA). This cell line was stably transfected with the luciferase reporter gene and estrogen-responsive element derived from the *Xenopus* vitellogenin A2 gene. ER agonists can induce the production of luciferase in MVLN cells (Pons et al. 1990). Cells were seeded in the interior 60 wells of a 96-well ViewPlate (Packard Instrument Company, Boston, MA, USA) at a density of 7 × 10^4^ cells/well, starved in SF medium for 48 hr, and exposed to test compounds for 2 days. A concentration range of 0.5 pM–1 nM E_2_ was used as a positive control, whereas the exposure concentration range of test chemicals was 0.1–100 μM. Luciferase activity was measured with the LucLite kit (Packard Instruments) according to the manufacturer’s protocol. We measured luminescence by microplate reader (Varioskan Flash) and integrated the luminescence signal for 10 sec. Total protein content was measured by the Bradford assay (Tiangen, Beijing, China) to normalize luminescent units. The results are given as relative luminescent unit per microgram protein. The maximal induction of positive control (corrected for solvent control, 0.1–0.2% ethanol) was set as 100%, and the responses of other chemicals were converted to a percentage of the maximum level. Three replicates were used in each experiment. The cytotoxicity of tested chemicals was examined by WST-1 kit in parallel and routinely observed under microscope to identify the exposure concentration range.

*RNA isolation and reverse transcription polymerase chain reaction (RT-PCR).* MCF-7BUS cells were seeded in six-well plates at the density of 1.0 × 10^6^ cells per well, starved in SF medium for 48 hr, and exposed to test compounds for 48 hr. First, cells were rinsed twice with cold phosphate-buffered saline, and total RNA was isolated using Trizol reagent (Invitrogen Inc., Carlsbad, CA, USA) following the manufacturer’s protocol. The 260 nm and 280 nm absorbance reading of total RNA was performed using a Nanodrop spectrophotometer (Thermo Scientific, Waltham, MA, USA). The concentration of RNA was quantified by the reading at 260 nm. The 260:280 nm ratios were between 1.8 and 2.0, which indicates that the extracted RNA was sufficiently pure.

We used a two-step quantitative RT-PCR to quantify gene expression. Total RNA was converted to cDNA using M-MLV (Moloney murine leukemia virus) reverse transcriptase (Promega, Madison, WI, USA) with oligo dT(15), following the manufacturer’s instructions. The final cDNA solution was diluted five times with DNase/RNase-free water (Gibco). Quantitative PCR was performed with a Stratagene MX3005 thermal cycler (Stratagene, La Jolla, CA, USA). PCR reaction mixtures (25 μL) contained 12.5 μL GoTaq Green Master Mix (Promega), 2 μL diluted cDNA, and 0.2 μM sense/antisense primers. The thermal cycle was 5 min at 95°C, followed by 45 cycles of 15 sec at 95°C, 30 sec at 55°C, and 30 sec at 72°C. The primer sequences of early growth response protein 3 (*EGR3*) were derived from Terasaka et al. (2004). We designed the primers of internal gene β*-actin* and trefoil factor 1 (*TFF1*; pS2) with Primer Premier 5 software (Premier Biosoft International, Palo Alto, CA, USA). The primer sequences were as follows: for β*-actin* (NM_001101), 5´-CACTCT​TCCAGCCT​TCCTTCC-3´ (forward) and 5´-AGGTCTTTGCG​GATGTCCAC-3´ (reverse); for *EGR3* (NM_004430), 5´-CCATGAT​TC​CTGACTACAACCTC-3´ (forward) and 5´-GTGGA​TCTGCT​TGTCTT​TGAATG-3´ (reverse); and for *TFF1* (NM_003225), 5´-AGAAGCG TGTCTGAGGTGTC-3´ (forward) and 5´-GCAAATAAG​GGCTGCTGTT-3´ (reverse). The quantification of target gene expression was based on a comparative cycle threshold (Ct) value. We normalized the expression level of each target gene to its reference gene β*-actin*. The fold change of the target genes was analyzed by the 2^−ΔΔCt^ method (Livak and Schmittgen 2001). We used melting curve analysis and agarose gel electrophoresis to verify the correct PCR products.

*Statistical analysis.* All results are expressed as the mean ± SD. For statistical analysis, we used one-way analysis of variance (ANOVA) and Tukey’s multiple range test to assess the significance of mean differences. Difference was considered significant at a *p*-value ≤ 0.05.

The concentration–response analyses were performed with four-parameter logistic curve regression analysis according to the following formula:

*y* = minimum + (maximum – minimum) ÷ [1 + (*x*/EC_50_)^Hill slope^], [1]

where *y* is the response value, *x* is the log concentration of the test compound, and EC_50_ is the concentration that induces half of the maximum proliferation effect or luciferase activity.

EC_50_ values were calculated from this nonlinear regression model. EC_20_ were calculated as

EC*_x_* = [*x*/(100 – *x*)]^(1/Hill slope)^ × EC_50_, [2]

where *x* is 20% of the maximum effects, and the Hill slope and EC_50_ were calculated from Equation 1.

All statistical analyses were performed using Sigma Plot (version 10.0; Systat Software Inc., San Jose, CA, USA).

## Results

**Figure 1:**
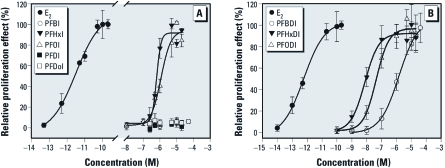
Concentration–response proliferation effects of FIAs (*A*) and FDIAs (*B*) in the E-screen assay. The relative proliferation effects are expressed as mean ± SD of triplicate measurements in one representative experiment.

*Stimulation of MCF-7 cell proliferation.* We used the E-screen assay to investigate the estrogenic activities of 12 PFIs in MCF-7 cells. FIAs are monoiodized fluorinated alkanes with even-numbered chains that have 4–12 carbons. PFBI [4 carbons in its alkyl chain (C-4)], PFDI (C-10), and PFDoI (C-12) did not show proliferation effects within the concentration ranges, whereas PFHxI (C-6) and PFOI (C-8) produced full concentration–response curves compared with E_2_ (Figure 1A); EC_50_ values were 0.63 μM and 1.15 μM for PFHxI and PFOI, respectively (Table 2). The proliferation effects appeared to be dependent on the chain lengths of FIAs. Fluorinated diiodine alkanes (FDIAs) have even-numbered chains with 4 to 8 carbons. All FDIAs produced full concentration–response curves in the E-screen assay (Figure 1B). The relative proliferation effects were in the following order: PFHxDI (C-6) > PFODI (C-8) > PFBDI (C-4). Likewise, proliferation potency also seems to be related to the specific carbon chain length of FDIAs. The EC_50_ values of PFBDI (1.45 μM), PFHxDI (7.5 nM), and PFODI (43.3 nM) were much lower than those of the corresponding FIAs with the same chain length. The order of their relative proliferation potencies was PFHxDI > PFODI > PFHxI > PFOI > PFBDI. These compounds are thus considered to behave like xenoestrogens in the E-screen assay.

**Table 2:** Maximum induction and effective concentrations of tested chemicals based on the E‑screen and MVLN assays.

E-screen					MVLN assay						
Compound	Maximum induction (%)*a*	EC_50_	Relative potency*b*	Maximum induction (%)*c *	EC_50_	EC_20_	Relative potency*b *
E_2_		100		3.12 pM		1		100		16.4 pM		4.14 pM		1
PFBI		ND*d*		—		—		ND*e*		—		—		—
PFHxI		99		0.63 μM		4.9 × 10^–6^		47		26.7 μM		14.1 μM		0.29 × 10^–6^
PFOI		101		1.15 μM		2.7 × 10^–6^		25		35.1 μM		20.4 μM		0.2 × 10^–6^
PFDI		ND*d*		—		—		ND*e*		—		—		—
PFDoI		ND*d*		—		—		ND*e*		—		—		—
4:2 FTI		ND*d*		—		—		ND*e*		—		—		—
6:2 FTI		ND*d*		—		—		ND*e*		—		—		—
8:2 FTI		ND*d*		—		—		ND*e*		—		—		—
10:2 FTI		ND*d*		—		—		ND*e*		—		—		—
PFBDI		97		1.45 μM		2.2 × 10^–6^		21		29.6 μM		13.8 μM		0.3 × 10^–6^
PFHxDI		100		7.5 nM		4.2 × 10^–4^		73		1.13 μM		0.38 μM		1.1 × 10^–5^
PFODI		105		43.3 nM		7.2 × 10^–5^		38		2.87 μM		1.07 μM		3.8 × 10^–6^
PFOA		ND*d*		—		—		ND*e*		—		—		—
PFOC		ND*d*		—		—		ND*e*		—		—		—
PFOB		ND*d*		—		—		ND*e*		—		—		—
1-Iodohexane		ND*d*		—		—		ND*e*		—		—		—
ND, not detected. **a**Percentage of the maximum proliferation effects of tested compounds to that of E_2_. **b**Ratio of EC_50_ of E_2_ to that of test compounds. **c**Percentage of the maximum induction effects of tested compounds to that of E_2_. **d**The maximum relative proliferation effect was < 7%; the absorbance ratio of E_2_ (100 pM) to control was 2.1 ± 0.2. **e**The maximum relative luciferase activity was < 5%; the relative luminescent unit ratio of E_2_ (1 nM) to control was 13.5 ± 2.4.														

*Transactivation in MVLN cells.* The MVLN assay has been widely used to study ER activity of test compounds (Freyberger and Schmuck 2005). In the present study, we used the MVLN assay to further investigate ER activity and estrogenic potency of PFIs. Before the MVLN assay, we tested the cytotoxic effects of each compound using the WST-1 assay. Exposure of MVLN cells to FIAs, FTIs, or FDIAs did not produce significant cytotoxicity within the concentration ranges, and no cytotoxic effects were observed by microscopic examination (data not shown).

**Figure 2:**
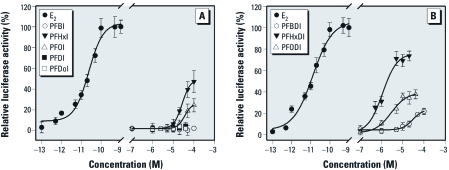
Concentration–response luciferase activity of FIAs (*A*) and FDIAs (*B*) in the MVLN assay. The relative luciferase activities are expressed as mean ± SD of triplicate measurements in one representative experiment.

Because these compounds did not show maximum induction compared with E_2_, relative potency based on the EC_20_ would be more reliable than that derived from the EC_50_ (Villeneuve et al. 2000). The estrogenic effects of PFIs revealed by the MVLN assay were in accordance with the results of the E-screen assay. As shown in Figure 2A, the induction of luciferase activity by PFBI, PFDI, and PFDoI were at the basal level (< 5%), whereas PFHxI and PFOI induced luciferase activity in a dose-related manner. PFHxI (EC_20_ = 14.1 μM) showed higher estrogenic activity than did PFOI (EC_20_ = 20.4 μM) in MVLN cells, with maximum induction values of 47% and 25%, respectively (Table 2). Luciferase activity induced by FDIAs seems to be related to the specific carbon chain length (Figure 2B). PFHxDI (EC_20_ = 0.38 μM) showed stronger estrogenic potency than did PFODI (EC_20_ = 1.07 μM) and PFBDI (EC_20_ = 13.8 μM), with the maximum induction values of 73%, 38%, and 21%, respectively. Because the difference of EC_50_ or EC_20_ values among PFHxI, PFOI, and PFBDI were small, we compared the estrogenic potency with the maximum induction value in an MVLN assay. The order of estrogenic potency was PFHxDI > PFHxI > PFODI > PFOI > PFBDI, which is comparable to results from the E-screen assay. Similarly, FDIAs possessed stronger estrogenic potency than did FIAs in the MVLN assay (PFBDI > PFBI; PFHxDI > PFHxI; PFODI > PFOI), which indicated that iodine substitution at the end of a fluorinated chain may enhance the estrogenic potency of FIAs. The optimum chain length for estrogenic activity was six carbons for FIAs and for FDIAs in both of these estrogen-screening assays.

*Comparison of PFCs with similar structures.* FTIs are partially fluorinated alkyl iodides, which are produced by the ethylation of FIAs in telomerization processes. Compared with FIAs and FDIAs, FTIs with various chain lengths did not show estrogenic effects in the E-screen or MVLN assays within the tested concentration ranges (0.01–200 μM). We used a nonfluorinated hydrocarbon, 1-iodohexane (C-6), as the control to study the effects of fluorination on estrogenic effects. Three eight-carbon PFCs—PFOA, PFOC, and PFOB—that contain no iodine substitution on the carbon chain were used as comparisons to study the effects of iodine substitution on estrogenic effects. As we suspected, 1-iodohexane, PFOA, PFOC, and PFOB showed negative results in the estrogen-screening assays (Table 2). These results further emphasize that a perfluorinated alkyl chain and iodine substitution are important structural features for the estrogenic effects of PFIs.

**Figure 3:**
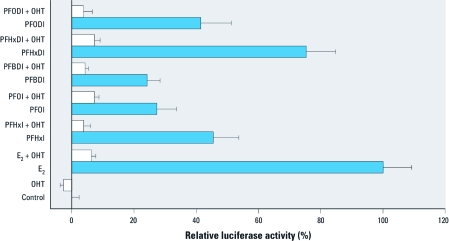
Coexposure effects of the tested chemicals with OHT in the MVLN assay. The ERα antagonist OHT (10 nM) was coexposed with 1 nM E_2_, 100 μM PFHxI, 100 μM PFOI, 100 μM PFBDI, 20 μM PFHxDI, or 40 μM PFODI. The relative luciferase activities are expressed as the mean ± SD of triplicate measurements in one representative experiment.

*Coexposure assay with OHT in MVLN assay.* We used OHT, a strong estrogen antagonist in the mammary gland, to block the ER in the MVLN assay. OHT was coexposed with PFHxI, PFOI, PFBDI, PFHxDI, or PFODI. We used the highest induction concentrations obtained from MVLN assay in the coexposure experiments and the gene expression assay. As shown in Figure 3, coexposure of OHT with the tested chemicals resulted in marked reduction of luciferase activity, which further confirmed that these xenoestrogens can activate the ER.

**Figure 4:**
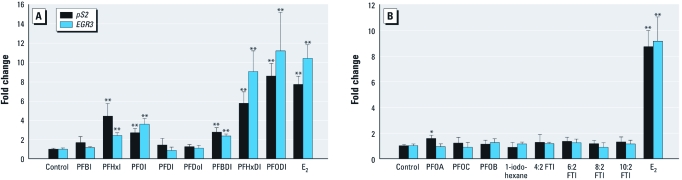
Effects of tested chemicals on mRNA expression of estrogen-responsive genes *TFF1* (pS2) and *EGR3 *in MCF-7 cells. (*A*) Cells exposed to 0.1% ethanol (control), 50 μM PFBI, 50 μM PFHxI, 50 μM PFOI, 40 μM PFDI, 40 μM PFDoI, 50 μM PFBDI, 20 μM PFHxDI, 40 μM PFODI, or 100 pM E_2_ for 48 hr. (*B*) Cells exposed to 0.1% ethanol, 50 μM PFOA, 50 μM PFOC, 50 μM PFOB, 50 μM 1-iodohexane, 50 μM 4:2 FTI, 50 μM 6:2 FTI, 40 μM 8:2 FTI, 40 μM 10:2 FTI, or 100 pM E_2_ for 48 hr. Results are expressed as the mean ± SD of triplicate measurements in one representative experiment. *p < 0.05, and **p < 0.01, compared with the control. ANOVA and Tukey’s multiple range test were used to assess the significance of mean differences.

*Expression of estrogen-responsive genes.* After MCF-7 cells were exposed to a series of PFCs for 48 hr, the expression levels of two estrogen-responsive genes (*EGR3* and *TFF1*) were analyzed by real-time PCR. The *TFF1* gene is involved in cell proliferation and also serves as a biomarker gene responding to estrogens (Brown et al. 1984; Jorgensen et al. 2000). As one of the ER-mediated estrogen-inducing genes, *EGR3* belongs to the early growth response family and plays an important role in the estrogen-dependent induction of the immune evasion system (Inoue et al. 2004). The expression levels of *EGR3* and *TFF1* are up-regulated by natural and synthetic estrogens in MCF-7 cells (Terasaka et al. 2004). PFHxI, PFOI, PFBDI, PFHxDI, and PFODI, which showed estrogenic effects in the E-screen and MVLN assays, significantly up-regulated the estrogen-responsive genes by 4.4-, 2.7-, 2.7-, 5.7-, 8.5-, and 7.7-fold for *TFF1* and by 2.4-, 3.6-, 2.4-, 9.1-, and 11.2-fold for *EGR3* (Figure 4A). PFOC, PFOB, 1-iodohexane, PFBI, PFDI, PFDoI, and FTIs did not affect the expression of *EGR3* or *TFF1*, but PFOA slightly up-regulated *TFF1* by 1.57-fold. The levels of *EGR3* and *TFF1* mRNA were greatly elevated by 8.7- and 9.2-fold upon exposure to E_2_ (Figure 4B). Therefore, PFHxI, PFOI, PFBDI, PFHxDI, and PFODI showed estrogenic activity in these assays. These xenoestrogens activated ER, which was followed by increased expression of the estrogen-responsive genes. PFBDI showed weaker estrogenic potency than did PFHxDI or PFODI in the E-screen and MVLN assays, whereas the expression of *EGR3* and *TFF1* induced by PFHxDI and PFODI was much higher than that induced by PFBDI. The up-regulation of *EGR3* and *TFF1* was also greater for FDIAs compared with the monoiodized FIAs. This adds further evidence that an increase in iodine substitution at the end of the fluorinated chain can enhance the estrogenic potencies of FIAs.

## Discussion

The endocrine-disrupting effects elicited by industrial chemicals have been of extensive concern (Colborn et al. 1993). Exposure to xenoestrogens may lower sperm count and male fertility and increase the incidence of breast and testicular cancer in humans (Toppari et al. 1996). Most of the adverse effects of these compounds are thought to be mediated through ER activation. Although the environmental behaviors of PFIs are not known, these volatile and high-production-volume chemicals could be released into the ambient environment during production, storage, and transport. The atmospheric oxidation of PFIs may contribute to the increased levels of other PFCs in the environment. Studies of the potential toxicities of PFIs are therefore needed for health risk evaluation. In this study, we investigated the estrogenic effects of PFIs by the E-screen and MVLN assays and the expression of estrogen-responsive genes. Our results showed that PFHxI, PFOI, PFBDI, PFHxDI, and PFODI exert estrogenic effects through activation of the ER. The relative estrogenic potencies obtained from the E-screen and MVLN assays are both related to the specific carbon chain length of FIAs and FDIAs. The optimum chain length for estrogenic effects is six carbons, and iodine substitution on the perfluorinated chain was crucial for the estrogenic effects. Those potent compounds were able to fully stimulate cell proliferation of MCF-7 cells, but this was not the case for the induction of reporter gene expression in MVLN cells. This discrepancy might be due to the difference of initial seeding density, exposure time, and sensitivity between the two assays. The expression of the estrogen-responsive gene by these PFIs further confirmed the results. The estrogenic potencies of FDIAs were higher than that of the FIAs, indicating that the increasing number of iodine substitutions on FIAs renders the chemical more potent in inducing estrogenic activity. PFHxDI (C-6), with two iodine substitutions (one at each end) of the perfluorinated chain, showed the highest potency among the PFIs.

Considerable evidence has indicated that chain length determines the biological effect of PFCs (Hu et al. 2002; Liao et al. 2009; Upham et al. 1998). Bioconcentration and bioaccumulation of PFCs are related to the length of the fluorinated chain in different species (Martin et al. 2003). Cytotoxic end points of PFCs such as *in vitro* cytotoxic effects, the alteration of cell membrane potential, and cytosolic pH are directly related to perfluorinated chain length (Kleszczynski and Skladanowski 2009). The inhibition of perfluorinated fatty acids on gap junction intercellular communication also depends on chain length; shorter PFCs, including perfluorobutanesulfonate and perfluorohexanesulfonate, did not show effects, whereas PFOS significantly inhibits gap junction intercellular communication (Hu et al. 2002; Upham et al. 1998). The interference of PFCs on cultured rat hippocampal neurons was also related to the carbon chain length and functional groups (Liao et al. 2009). Our findings suggest that FDIAs and some of the FIAs exert estrogen effects through the activation of ER. Because the solubility of nonpolar FIAs in culture media decreased with increasing chain length, the lack of estrogenic effects for PFDI and PFDoI might be attributed, in part, to decreased solubility and bioavailability of long-chain FIAs.

We used the nonfluorinated organic iodide 1-iodohexane to study the effect of fluorination on estrogenic effects compared with PFHxI. In the screening assays, 1-iodohexane did not exert estrogenic effects, indicating that fluorination is an important structural feature for estrogenic activity. The hydrophobic property of the fluorinated chain imparts the proteinophilic and lipophilic property of PFCs and results in the interaction of PFCs with multiple biological molecular targets in various species. PFOA did not show proliferation effects in MCF-7 cells, as previously reported by Maras et al. (2006). In the present study, we found that PFOA, PFOB, and PFOC also lack estrogenic effects. By comparing the structure–activity relationship between these PFCs, we propose that the iodine substitution is a key attribute for the estrogenic effect. The estrogenic effect was also lower for monoiodized fluorinated alkanes than for diiodized fluorinated alkanes, which further supports our assumption.

FTOHs exert estrogenic activity in MCF-7 cells and aquatic organisms (Ishibashi et al. 2008; Maras et al. 2006). FTOHs behave as estrogens because of the similarity of their chemical structure and properties to other xenoestrogens, such as 4-nonylphenol. In the telomerization processes, fluorotelomer iodides are oxidized to produce FTOHs. Compared with FTOHs, none of the FTIs induced cell proliferation, which indicated that the hydroxyl group is more important for the estrogenic effects than is iodine substitution in partially fluorinated chemicals. Some of the PFIs activated the ER and induced luciferase activity in MVLN cells. However, it is questionable whether these PFIs are able to directly bind to and activate the ER. Structural features such as a phenol ring and a hydrophobic group attached *para* to the hydroxyl group are essential for the estrogenic effects (Blair et al. 2000; Laws et al. 2006; Suzuki and Shapiro 2007). Furthermore, hydroxylated analogs of polybrominated diphenyl ethers and polychlorinated biphenyls have been shown to exert estrogenic effects (Bergeron et al. 1994; Fielden et al. 1997; Meerts et al. 2001). Therefore, it may be reasonable to expect that hydroxylated forms of FIAs and FDIAs could also be estrogenic.

Compared with PFOI, both PFOB and PFOC showed no estrogenic activity. It is likely that bond strength also determines their reactivity. The strength of the bonds is C–F (467 kJ/mol) > C–H (453 kJ/mol) > C–Br (290 kJ/mol) > C–I (228 kJ/mol). Among the four halogens, fluorine is the most electronegative and iodine the least. The polarization of the C–I bond is lower than that of the C–H bond and the other carbon–halogen bonds. Because iodine is a good leaving atom and because of the chemical reactivity of the C–I bond, it would be easier for PFIs to be converted to their hydroxylated analogs during the exposure studies. Oxidation of FIAs can result in the formation of PFCAs (Lehmler 2005). In this reaction, C*_n_*F_2_*_n_*_+1_OH is thought to have been formed by the cleavage of C–I bonds in FIAs and addition of OH (Yamamoto et al. 2007). We hypothesize that C*_n_*F_2_*_n_*_+1_I is hydrolyzed to C*_n_*F_2_*_n_*_+1_OH in the culture media or inside the cells, and the degradation products or the metabolites of PFIs are the possible targets for ER, thereby exerting estrogenic activity. However, the underlying mechanisms for the estrogenic effects of PFIs have not been completely clarified, and further studies are also warranted to characterize possible catabolites of PFIs, which might also exhibit estrogenic activity.

The main functions of hormones are to maintain homeostasis and regulate reproduction and development. Exposure to endocrine-disrupting chemicals may cause adverse effects to the organs and glands that secrete hormones, further resulting in endocrine toxicity such as impaired reproduction and development. PFIs are volatile chemicals and have been detected around fluorochemical manufacturing areas (Ruan et al. 2010a). As important precursors for the synthesis of organic fluoride products, PFIs could be incorporated into fluorotelomer raw materials and fluorotelomer-based products as residues (Larsen et al. 2006). Occupational and indoor environments might be exposure risk zones, and inhalation could be a possible exposure route.

## Conclusion

Some PFIs could act on ERs and potentially cause detrimental effects on reproductive and developmental systems. To our knowledge, this is the first study to find estrogenic activity of PFIs using three *in vitro* methods. Considering the current large and increasing production volume of telomerization-based PFCs, more extensive studies should be conducted on the environmental distribution and toxicological effects of PFIs.
